# Constitutive and regulated expression vectors to construct polyphosphate deficient bacteria

**DOI:** 10.1186/1756-0500-2-50

**Published:** 2009-03-26

**Authors:** Francisco P Chávez, Cecilia Mauriaca, Carlos A Jerez

**Affiliations:** 1 Laboratory of Molecular Microbiology and Biotechnology & Millennium Institute for Advanced Studies in Cell Dinamics and Biotechnology (ICDB), Department of Biology, Faculty of Sciences, University of Chile, Las Palmeras 3425, Ñuñoa, Santiago, Chile

## Abstract

**Background:**

Inorganic polyphosphate (polyP), a polymer of tens or hundreds of phosphate residues linked by ATP-like bonds, is found in all organisms and performs a wide variety of functions. PolyP is synthesized in bacterial cells by the actions of polyphosphate kinases (PPK1 and PPK2) and degraded by an exopolyphosphatase (PPX). Bacterial cells with polyP deficiencies are impaired in many structural and important cellular functions such as motility, quorum sensing, biofilm formation and virulence. Knockout mutants of the *ppk1 *gene have been the most frequent strategy employed to generate polyP deficient cells.

**Results:**

As an alternative method to construct polyP-deficient bacteria we developed constitutive and regulated broad-host-range vectors for depleting the cellular polyP content. This was achieved by the overexpression of yeast exopolyphosphatase (PPX1). Using this approach in a polyphosphate accumulating bacteria (*Pseudomonas sp*. B4), we were able to eliminate most of the cellular polyP (>95%). Furthermore, the effect of overexpression of PPX1 resembled the functional defects found in motility and biofilm formation in a *ppk1 *mutant from *Pseudomonas aeruginosa *PAO1. The plasmids constructed were also successfully replicated in other bacteria such as *Escherichia coli, Burkholderia *and *Salmonella*.

**Conclusion:**

To deplete polyP contents in bacteria broad-host-range expression vectors can be used as an alternative and more efficient method compared with the deletion of *ppk *genes. It is of great importance to understand why polyP deficiency affects vital cellular processes in bacteria. The construction reported in this work will be of great relevance to study the role of polyP in microorganisms with non-sequenced genomes or those in which orthologs to *ppk *genes have not been identified.

## Background

Polyphosphate (polyP) is a ubiquitous linear polymer of hundreds of orthophosphate residues (Pi) linked by "high-energy" phosphoanhydride bonds. The best-known enzymes involved in the metabolism of polyP in bacteria are the polyphosphate kinases (PPKs) that catalyze the reversible conversion of the terminal phosphate of ATP (or GTP) into polyP and the exopolyphosphatase (PPX) that processively hydrolyzes the terminal residues of polyP to liberate Pi [1,2].

The involvement of polyP in the regulation of both, enzyme activities and the expression of large groups of genes is the basis for the survival of different bacteria, including pathogens, to stressing conditions and during adaptation to the stationary phase of growth [2,3]. PPK1 knockout mutant cells lacking polyP survive poorly during growth in the stationary phase and are less resistant to heat, oxidants, osmotic challenge, antibiotics and UV [4-8]. Important cellular process such as motility, quorum sensing, biofilm development and virulence are also affected [9-11]. In addition to homologues of PPK1, another widely conserved polyP enzyme is PPK2 [12,13]. In contrast to the ATP-dependent polyP synthetic activity of PPK1, PPK2 preferentially catalyses the opposite reaction, i.e. poly P-driven synthesis of GTP from GDP. Orthologs to both proteins have been found in many bacterial genomes. Some bacteria possess orthologs of either PPK1 or PPK2, or both, or neither. For example, *E. coli *has only PPK1 and *Pseudomonas aeruginosa *PAO1 contains both. Interestingly, the enzyme in charge of polyP synthesis still remains unknown in several bacteria containing the biopolymer [13].

As a tool to remove cellular polyP content, we report here the construction of expression vectors with constitutive and regulated promoters that overexpress in bacteria the yeast PPX1 fused to a hexa histidine-tag (6 Ht). Both, constitutive and inducible expression of PPX1 removed almost all cellular polyP (>96%) in polyP-accumulating *Pseudomonas sp*. B4. The absence of the biopolymer was confirmed by transmission electron microscopy (TEM) and by quantification using a two-step method [14]. Recombinant polyP-deficient cells resembled *ppk1 *mutants of many bacteria in their functional defects found in motility and biofilm formation. However, these vectors have a greater advantage compared with previously described methods since they could be used in bacteria with unknown genome sequences or in those for which *ppk *orthologs have not been found. Furthermore, the regulated expression of PPX1 allowed us to induce polyP deficiency when required. Importantly, the vectors used in this work have been successfully replicated in other bacteria from the *Escherichia*, *Salmonella, Pseudomonas *and *Burkholderia *genus demonstrating the wide utility of this approach.

## Results

**Figure 1:**
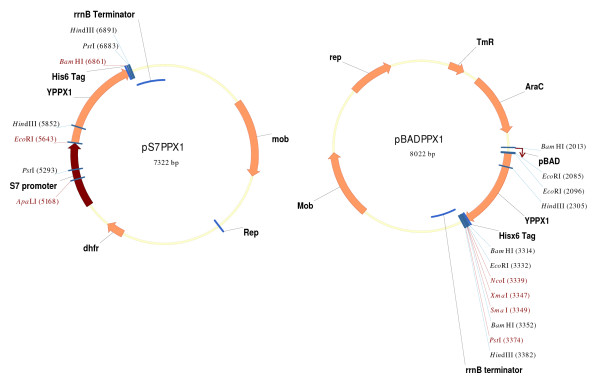
**Broad-host range constitutive (pS7PPX1) and regulated (pBADPPX1) expression vector maps to obtain polyP deficient bacteria**. Source of yeast DNA and primers used in plasmid construction were described in Methods. A detail description of pMLBAD and pMLS7 plasmids can be found in reference [22].

**Figure 2:**
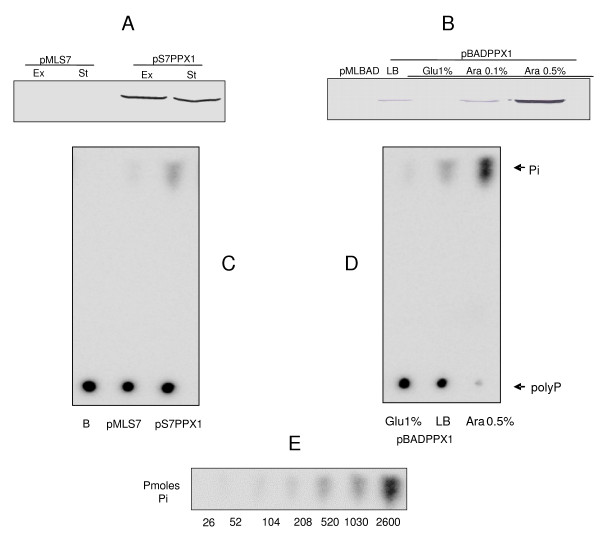
**Constitutive (pS7PPX1) and regulated (pBADPPX1) overexpression of yeast *PPX1 *in *Pseudomonas sp*. B4 cells**. (A, B) Westtern-blotting using anti His6t mAb and (C, D) thin layer chromatography (TLC) of exopolyphosphatase activity products. (E) Phosphate (Pi) standard curve to determine exopolyphosphatase activity. Cells were from exponential (Ex) or stationary (St) phases.

Details about the construction of the constitutive and regulated expression vector and the verification of yeast PPX1 overexpression can be found in the Additional File 1 and Figures 1 and 2.

### Polyphosphate content in recombinant *Pseudomonas sp*. B4 cells

**Figure 3:**
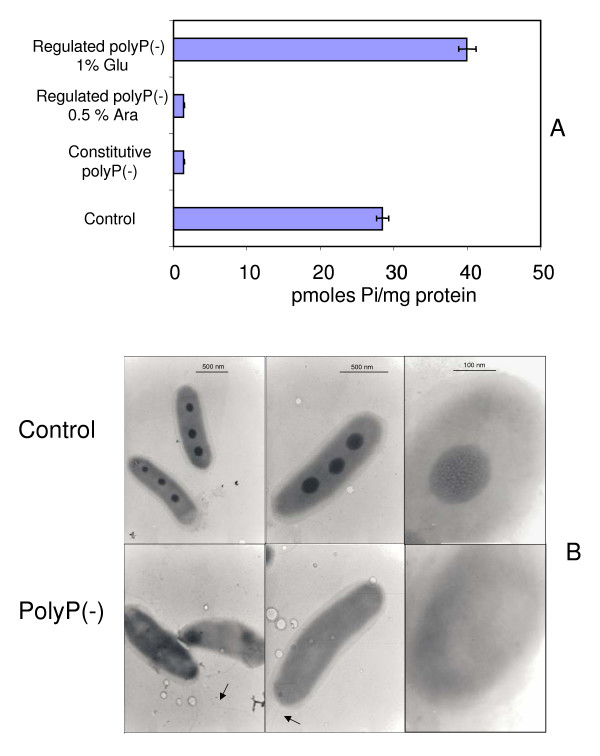
**PolyP content in recombinant *Pseudomonas sp*. B4 cells**. PolyP removal was checked by quantification of cellular polyP (A) and by Transmission Electron Microscopy (TEM) of unstained cells (B). Arrows indicate the cellular flagellum. Error bars are the average of three determinations in three biological replicates.

Previous studies from our laboratory have shown that *Pseudomonas sp*. B4 is a polyP-accumulating bacteria and in certain conditions polyP can be stored and observed as electron dense granules by transmission electron microscopy (TEM) of unstained cells [15,16]. To determine whether the overproduction of yeast PPX1 (Figures 1 and 2) affected the accumulation of polyP in the bacterium, we monitored the levels of this biopolymer by observing the presence of electron dense granules by TEM. Figure 3A shows the results of quantification of the isolated polyP from recombinant *Pseudomonas sp*. B4 cells in LB medium. PolyP from arabinose induced regulated polyP(-) (pBADPPX1) and constitutive polyP(-) (pS7PPX1) cells was removed to barely detectable levels (around 2 pmoles Pi/mg protein). The reduction of polyP levels (>96%) due to PPX1 overproduction could be prevented in regulated polyP(-) (pBADPPX1) cells to levels similar to those of the control cells (pMLBAD and pMLS7) when growing in the presence of 1% glucose.

PolyP in the form of electron dense granules can be seen when *Pseudomonas sp*. B4 is grown in the presence of biphenyl in minimal medium (M9) [15,16] (Figure 3B, control). Nevertheless, these granules were not present in constitutive polyP(-) (pS7PPX1) cells (Figure 3B). Quantification of polyP in these samples confirmed the removal of almost all cellular polyP (data not shown). These findings clearly show that overexpression of PPX1 is an excellent method to remove cellular polyP.

### Functional analysis of constitutive and inducible polyP deficient *Pseudomonas sp*. B4 cells

**Figure 4:**
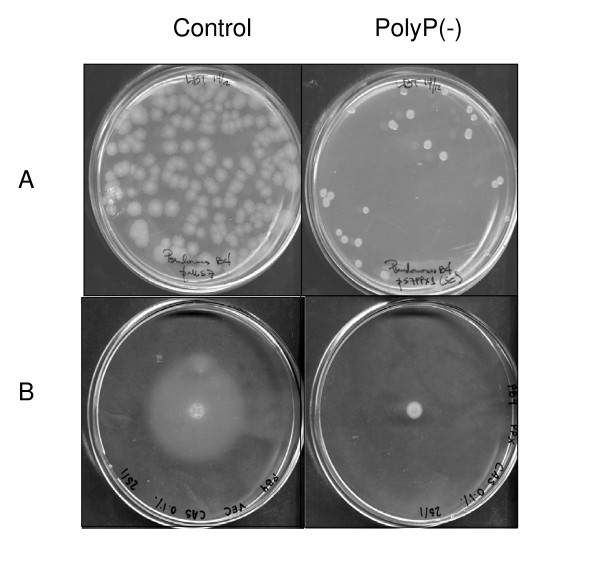
**Colony morphology and motility of constitutive polyP deficient *Pseudomonas sp*. B4 cell**. (A) Standard LB plates and (B) motility assay plates of control and polyP deficient *Pseudomonas sp*. B4 cells.

Among many functional and structural problems, *P. aeruginosa *PAO1 *ppk1 *knockout mutants failed to develop biofilms and were impaired in all forms of motility (swimming, swarming, and twitching) [9-11,17]. To validate our approach and check whether our transformants resembled the functional deficiencies of *ppk1 *mutants, we performed motility and biofilm assays in our strains with depleted polyP levels. Wild type *Pseudomonas sp*. B4 is a highly motile rod with a single polar flagellum and forms widely spread colonies in LB plates [16]. However, the form and size of the colonies varied notoriously in constitutive polyP(-) cells, suggesting a motility defect (Figure 4A). This was confirmed by using semisolid agar plates where cells were able to swim through water-filled channels to create concentric chemotactic rings (Figure 4B). As reported for the *P. aeruginosa *PAO1 *ppk1 *mutant, our constitutive polyP(-) cells were impaired in swimming motility in semisolid agarose plates (Figure 4B) despite possessing an apparently normal flagellum (Figure 3 for flagellum detail). Curiously, the phenotype change seen in Figure 4 is as drastic as that seen in a nonmotile strain containing a knockout mutation in a flagellin structural gene *fliC *and more severe than that observed in a *ppk1 *mutant [9]. This suggests that the degree of lack of motility is related to the extent of polyP removed from the cell.

**Figure 5:**
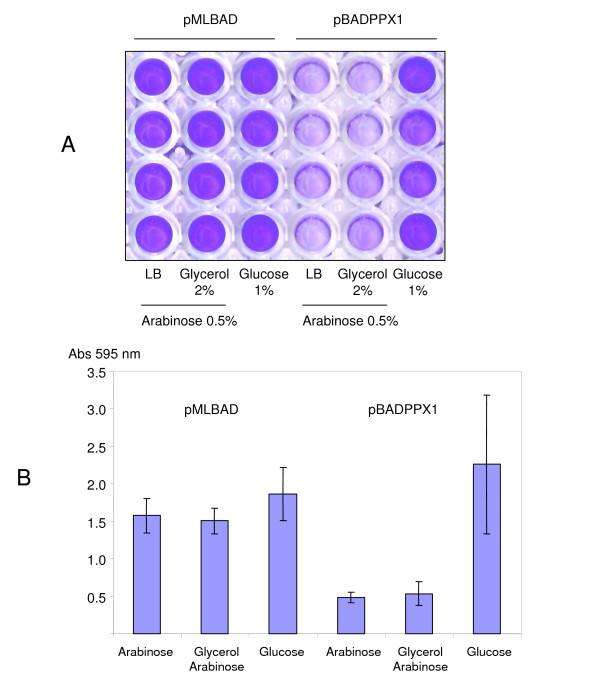
**Biofilm formation assays in regulated polyP-deficient *Pseudomonas sp*. B4 cells**. (A) Crystal Violet stained PVC plates of polyP-deficient *Pseudomonas sp*. B4 cells under repressed (Glucose 1%) and induced (Arabinose 0,5%,) conditions. (B) Quantification of crystal violet dye attached to the cells forming biofilms on PVC plates. Error bars are the average of two determinations in four biological replicates.

Finally, we measured the capacity of our regulated polyP(-) strains to attach and form biofilms on an abiotic surface in a simple assay. As shown in Figure 5, polyP deficient cells were able to form biofilm only when PPX1 expression was repressed in glucose. Just as in the case of the *ppk1 *mutants, our recombinant polyP(-) cells failed to attach to the inert surface when growing in the presence of arabinose (induction condition).

## Discussion

The results from this study demonstrate the usefulness of expression vectors for producing polyphosphate deficient bacteria. As far as we know, this strategy was employed only once in *E. coli *by using a very high copy number plasmid with a potent promoter [18]. Overexpression of recombinant proteins from multiple-copy plasmids can result in expression levels of up to 40% of the total cell protein. However, in most cases, this results in the formation of insoluble protein aggregates known as inclusion bodies, which may affect the activity of the expressed proteins [19,20]. In our case, plasmids pMLS7 and pMLBAD are derivatives of the pBBR1 plasmid [21], originally isolated from *Bordetella bronchiseptica*, which is maintained at around 20 to 30 copies per cell. This feature allowed us both, to actively express yeast PPX1 and to avoid affecting the general translation process in the host cell.

To our knowledge this is the first report of polyP-deficient bacteria being generated by constitutive and regulated low copy number vectors. The use of this approach allows the researcher to avoid using knockout mutants, which is currently the most commonly used procedure to diminish polyP content in bacteria. This is important for several reasons. First, the generation of knockout mutants requires the knowledge of the PPK sequence to be deleted and in the cases of bacteria with unknown genome sequences, this requires the isolation, cloning and sequencing of the corresponding gene. Second, in many bacteria there is more that one *ppk *gene responsible for polyP synthesis in the cell [12,17]. As a consequence, to abolish most of the polyP in these bacteria, more than one deletion would be necessary. Finally, despite the presence of polyP, in many bacteria no *ppk *orthologs have been found in their genomes. For this kind of bacteria our approach is the only reliable method to generate polyP deficiency. Even more, considering that the enzymes in charge of polyP synthesis are still unknown in *Archaea *and Eukarya, overexpression of PPX1 can be an effective method to produce polyP scarcity in these domains of life. We have demonstrated in this study that, regardless of the number of *ppk *genes or their equivalents present in a genome, the overexpression of yeast PPX1 eliminates almost all cellular polyP contents.

Knockout mutants are very sable throughout generations and one possible disadvantage of our method compared to the deletion strategy could be the plasmid stability in the transformants. Nevertheless, despite strain variations, these plasmids remain relatively stable for a number of generations in the absence of selection (88% in *Burkholderia cepacia *and 92% in *Escherichia coli*) [22]. In the case of *Pseudomonas sp*. B4 plasmid stability was around 90% (data not shown) but lower levels of stability might be found in other strains depending on their expression levels of Rep protein, some host-specific effects on plasmid partition or due to incompatibility with indigenous plasmids.

Another important aspect of the present construction is the use of a tag (His6t) that allows to follow up easily the expression of *PPX1 *during the experiments.

We have also established that the arabinose-inducible/glucose-repressible promoter permits to design experiments where polyP can be depleted when required. This approach will help to clarify the role of polyP in bacterial pathogenesis and other important microbial functions.

Altogether, these results demonstrate that overexpression of PPX1 generates functional defects similar to those previously described in *ppk1 *mutants. However, the use of the new constructions reported here constitutes an advantageous alternative method to study polyP deficiency in bacteria possessing more than one *ppk *gene or in those in which the enzymatic activity synthesizing polyP is unknown.

## Conclusion

PolyP has numerous and varied biological functions that have been discovered mainly by studying its deficiency in bacteria. To better understand the function of polyP is necessary then to have simple approaches to eliminate this biopolymer in the cell. In this study we developed broad-host-range constitutive and regulated vectors that deplete cellular polyP. We conclude that these vectors will function as suitable and efficient genetic systems for characterizing polyP deficiency in bacteria, especially in those microorganisms with unknown genome sequences.

## Methods

### Bacterial strains, genomic DNA, plasmids and growth conditions

Bacterial strains and plasmids used in this study are listed in Table 1. *Escherichia coli *and *Pseudomonas *sp. B4 strains and their derivative strains were grown aerobically at 37°C on Luria-Bertani (LB) rich medium. Trimetropim (50 μg/ml) was added when required. *E. coli *was cultured in Luria-Bertani medium or on Luria-Bertani (LB) agar plates at 37°C. For the expression experiments the LB medium was supplemented with 1% (w/v) glucose or 0.1%–0.5% (w/v) arabinose as required. Genomic DNA from *Saccharomyces cerevisiae *W303 was used to amplify the *PPX1 *gene.

**Table 1:** Strains and plasmids used in this study

**Strains**	**Characteristics**	**Reference**
*Pseudomonas sp*. B4 constitutive polyP(-)	*Pseudomonas sp*. B4 transfomant with pS7PPX1 plasmid	This study
*Pseudomonas sp*. B4 constitutive control	*Pseudomonas sp*. B4 transfomant with pMLS7 plasmid	This study
*Pseudomonas sp*. B4 regulated polyP(-)	*Pseudomonas sp*. B4 transfomant with pBADPPX1 plasmid	This study
*Pseudomonas sp*. B4 regulated control	*Pseudomonas sp*. B4 transfomant with pMLBAD plasmid	This study
*Escherichia coli *NR100	M15 (pREP) derivative with pQE30PPK plasmid	[24]
**Plasmids**		
pGEM^®^-T-easy	T-vector cloning system	Promega
pMLBAD	Broad-host range regulated expression vector	[22]
pMLS7	Broad-host range constitutive expression vector	[22]
pTYPPX1	pGEM^®^-T-easy with *PPX1 *gene from *S. cerevisiae*. (His6 tag)	This study
pS7PPX1	pMLS7 with *PPX1 *gene from *S. cerevisiae*. (His6 tag)	This study
pBADPPX1	pMLBAD with *PPX1 *gene from *S. cerevisiae*. (His6 tag)	This study

### Polyphosphate methods

*PolyP quantification*. Purified recombinant His6-PPK was prepared by using *E. coli *strain NR 100 as described previously [23,24] and this preparation was used in the polyP assay described below. The protein concentration was determined by the method of Bradford (Coomassie Plus protein Assay, Pierce). PolyP was quantified by using a two-step conversion of polyP into ATP by PPK and quantification of ATP by using luciferase to generate light [14,25]. PolyP was extracted from small pellet cells by using Glassmilk. The resulting PolyP was assayed by using the reverse reaction of *E. coli *PPK in ADP excess. Finally, the ATP generated was determined by using the luciferase (Boehringer Mannheim) reaction, and the luminescence was measured with a luminometer (BioScan Lumi/96). Concentration of polyP was expressed in terms of Pi residues.

*Assay for PPX activity and TLC analysis of the reaction products*. First, radioactively labelled polyP with a chain length of 750 residues was prepared as previously described [14]. The identity and purity were determined by its susceptibility to hydrolysis by PPX1.

PPX activity was determined as previously described [26], with the following modifications. A 50 μl reaction mixture contained 50 mM Tris/acetate (pH 7.0), 1 mM MnCl_2_, 100 mM KCl, 50 μg extract protein and 250 μM [^33^P] polyP750. Reactions were stopped after incubation of the mixtures for 60 min at 65°C. After this, 4 μl was taken from each reaction mixture and loaded on polyethyleneimine-cellulose plates (Merck). For TLC, samples of 4 μl were separated in 0.75 M KH_2_PO_4 _(pH 3.5). Radioactive spots were visualized and quantified by using a Phosphorimager (Molecular Imager FX Systems, Bio-Rad). One unit of enzyme was defined as the amount releasing 1 pmol of phosphate from polyP min^-1^.

More details about the Methods employed in this work were included in the Additional File 1.

## Competing interests

The authors declare that they have no competing interests.

## Authors' contributions

FCH and CAJ conceived and designed the study. FCH performed the experiments and drafted the manuscript. CM carried out some experiments. CAJ participated in coordination and funding for the study, critical evaluation and amended the manuscript. All authors read and approved the final manuscript.

## Supplementary Material

Additional File 1 **Construction and characterization of constitutive and regulated expression vectors for generation of polyP-deficient bacteria.** Methods and Results. The data provided the methods for the construction of constitutive and regulated expression vectors to study polyP deficiency in Gram-negative bacteria as exemplified in the overexpression of exopolyphosphatase from yeast in the genus *Pseudomonas*. Click here for file
