# Supplementary material for: Constitutive and regulated expression vectors to construct polyphosphate deficient bacteria
Source: BMC Res Notes. 2009 Mar 26;2:50. doi: 10.1186/1756-0500-2-50 (PMC2667530; doi:10.1186/1756-0500-2-50)
Supplement: Additional File 1 — Construction and characterization of constitutive and regulated expression vectors for generation of polyP-deficient bacteria. Methods and Results. The data provided the methods for the construction of constitutive and regulated expression vectors to study polyP deficiency in Gram-negative bacteria as exemplified in the overexpression of exopolyphosphatase from yeast in the genus Pseudomonas. [file 1756-0500-2-50-S1.rtf]

Additional File 1
Construction and characterization of constitutive and regulated expression vectors for generation of polyP-deficient bacteria. Methods and Results Construction and characterization of constitutive and regulated expression vectors for generation of polyP-deficient bacteria. Methods and Results 
Results 
Construction of constitutive and regulated expression vectors to study polyP deficiency

To study polyP deficiency in bacteria, an alternative strategy to deletion of PPK genes is the overexpression of PPX. To ensure efficient expression in bacteria we employed constitutive (pMLS7) and regulated (pMLBAD) vectors. The choice of these vectors was based in their different features such as plasmid stability, copy number, selectable marker and cloning facilities. Previously constructed broad-host-range vectors pMLS7 and pMLBAD were successfully used as cloning and expression vectors in B. cepacia complex strains and Burkholderia isolates [1] as well as in other of its derivatives from nonenteric bacteria [2, 3]. Thus, to obtain recombinant bacteria with polyP deficiency we used the PPX1 gene from Saccharomyces cerevisiae fused to a His6 tag. A yeast gene was employed because unlike what is observed in bacteria [4], it is not inhibited by ppGpp and other related molecules and its activity can be also distinguished from the host exopolyphosphatases [5].
Yeast PPX1 gene was amplified by PCR and cloned in pGEM®-T-easy vector and the resulting plasmid (pTYPPX1) served as the backbone for the construction of both the constitutive and inducible expression vectors described in this work (pS7PPX1 and pBADPPX1, Figure 1). Both plasmids were mobilized into Pseudomonas sp. B4 by electroporation and shown to confer resistance to 100 μg/ml trimethoprim in LB plates. Colony clones were checked for the presence of PPX1 gene by PCR and their plasmids isolated and sequenced to check the integrity of PPX1 gene. Plasmids pMLS7 and pMLBAD were also transformed in Pseudomonas sp. B4 and used as controls in all experiments.
Overexpression of yeast PPX1 in Pseudomonas sp. B4 

To check the expression of PPX1 gene and to asses its functionality in Pseudomonas sp. B4, all transformants were subjected to three different assays: 1) Western blot with antibodies against His6 tag, 2) exopolyphosphatase activity in thin layer chromatography and 3) polyP content. 
The expression of the PPX1 protein was first assessed by Western blot analysis. As shown in Figure 2A the band intensity in samples prepared from constitutive polyP(-) cells (pS7PPX1) were constant in both exponential and stationary phases of growth, indicating the constitutive expression of PPX1 in this bacterium. As expected, no protein bands were visible in samples prepared from the control cells clones (pMLS7). On the other hand, Figure 2B shows that the amount of PPX1 produced by regulated polyP(-) cells (pBADPPX1) increased as a function of L-arabinose concentration in the culture medium. The sample obtained from cells grown in the absence of L-arabinose (Figure 2B, lane LB) showed a weak signal, suggesting that some component of the LB medium is able to produce a small amount of protein under the control of pBAD promoter. Similar results were obtained in Burkholderia strains when expressing eGFP [1]. As expected, no band was visible neither in samples from cells grown in glucose nor in control cells (pMLBAD). This confirmed that promoter activity was fully repressed in the presence of glucose. Overall, data from protein expression experiments conclusively demonstrated that both promoters were active in Pseudomonas sp. B4 and particularly that by using these vectors, PPX1 expression can be either constitutive or tightly controlled by the carbon source employed.
To assess whether PPX1 was functionally expressed in these strains and conditions, the exopolyphosphatase activity was quantified using thin layer chromatography (Figure 2C and D). It should be mentioned that the assayed PPX activity corresponds to the optimal conditions for the yeast enzyme and under those circumstances the background bacterial PPX activity is barely detected (data not shown). As shown in Figure 2C and D, the PPX activity pattern exactly matched the results obtained by western blot analysis; i.e. no activity was detected in samples with no cell-extract (B), in control cells (pMLS7) and in extracts from cells grown in glucose. Maximum activity was obtained in extracts from cells induced with L-arabinose. Thus, our data from western blot and PPX activity assays showed that in Pseudomonas sp. B4, PPX1 was efficiently expressed when carrying both the constitutive or inducible expression vectors constructed. 
Methods
Transformation by electroporation
Electrocompetent cells were prepared similarly for all bacterial species. An overnight culture grown in SOB medium (per liter: 20 g of tryptone, 5.0 g of yeast extract, 0.584 g of NaCl and 0.186 g of KCl, adjusted to pH 7.0 with NaOH) was diluted (1:1,000) in a total volume of 500 ml of SOB and grown to an optical density of 0.8 at 600 nm. Cells were harvested by centrifugation at 5,000 xg in a Sorvall GSA rotor at 4°C for 10 min. For washing, the cell pellet was resuspended in 500 ml of ice-cold 10% (vol/vol) glycerol and recentrifuged as described above for 15 min. The washing step was repeated. The pellet was subsequently resuspended in 10% glycerol (total volume, 2 ml). Cells aliquots were frozen in liquid nitrogen and stored at -80°C until thawed for electroporation.
DNA manipulation and vectors construction
To construct the expression vectors that overexpressed yeast PPX1 fused to His6 tag (H6t), first the yeast gene was amplified using the sequence specific primers 5'GGAATTCATGTCGCCTTTGAGAAAGAC3' and 5'CCGGATCCTCAATGATGATGATGATGATGCTCTTCCAGGTTTGAGTACGC3' containing restriction sites for EcoRI and BamHI, respectively. The 1,223 bp PCR product was purified by using SV Gel and PCR Clean-Up System (Promega, USA) and cloned in pGEM®-T-easy vector (Promega, USA). Orientation and integrity of the cloned gene were confirmed by sequencing both DNA strands in 4 selected clones.
For the construction of the regulated vector that overexpressed yeast PPX1 fused to His6 tag (H6t), pBADPPX1 (Figure 1) and plasmid pTYPPX1 were digested with EcoRI and BamHI, and directionally cloned downstream of the BAD promoter (E. coli araC-PBAD system) of EcoRI/BamHI-treated pMLBAD vector. In the case of the constitutive vector construction (pS7PPX1, Figure 1) plasmid pTYPPX1 was digested with EcoRI and cloned downstream of the Burkholderia xenovorans LB400 S7 promoter of EcoRI-treated pMLS7. Clones were selected for the correct orientation and all were confirmed by sequencing both DNA strands. Restriction endonucleases, T4 DNA ligase, polymerase, and nuclease were purchased from New England Biolabs (USA) and used according to manufacture's instructions.
Electron microscopy
Unstained cells from the different cultures were routinely examined for the presence of electron-dense bodies by transmission electron microscopy [6]. Cells from different strains were dispersed in distilled water and added onto carbon-coated nickel grids. The drops containing the microorganisms were drained off with filter paper and air dried during 30-50 s. Electron microscopy was performed with a Philips Tecnai 12 electron microscope using 80 kV accelerating voltage (Electron Microscopy Laboratory, Pontificia Universidad Católica de Chile).
Motility and biofilm assays
Swimming motility assays were performed as previously described [7], in plates with tryptone broth [per liter: 10 g tryptone (Difco) and 5 g NaCl] that contained 0.3% (wt/vol) agarose (GIBCO/BRL). Swim plates were inoculated with bacteria from an overnight culture in LB agar plates (1.5%, wt/vol) at 37°C with a sterile toothpick. The plates were then wrapped with Saran Wrap to prevent dehydration and incubated at 30°C overnight (12-14 h). Swarm plates media was basically the same used in swim plates but with 0.5% (wt/vol) Difco bacto-agar and plates were allowed to dry at room temperature overnight before being used. 
Biofilm assays were performed in 96 well plates as described earlier [6, 8]. The strains were cultured in LB medium in separate wells of a polystyrene microtiter dish followed by staining with crystal violet. Cell-attached dye was solubilized with 80% ethanol and the optical density was measured at 595 nm. When required media were supplemented with 1% of glucose (represor) or 0.5% arabinose (inducer). Since arabinose is not used by cells as a carbon source, 2% glycerol was present during the inducing conditions to avoid any effect due to the carbon source employed.
Western immunoblotting
Total proteins from different cells were separated by SDS-PAGE and electrotransferred to a PVDF membrane as already described [6]. Anti-Histidine-Tagged (H6t) Protein Mouse mAb (Novagen, USA) was employed (1:5,000 dilution) as the primary antibody and monoclonal anti-rabbit antibodies conjugated with peroxidase (Amersham, U.K.) as the secondary antibodies (1:5,000 dilution). A colorimetric method was used to develop western-blots as previously reported [6].
References
1.	Lefebre M, Valvano M: Construction and evaluation of plasmid vectors optimized for constitutive and regulated gene expression in Burkholderia cepacia complex isolates. Appl Environ Microbiol 2002, 68:5956-5964.
2.	Elzer P, Kovach M, Phillips R, Robertson G, Peterson K, Roop Rn: In vivo and in vitro stability of the broad-host-range cloning vector pBBR1MCS in six Brucella species. Plasmid 1995, 33:51-57.
3.	Sukchawalit R, Vattanaviboon P, Sallabhan R, Mongkolsuk S: Construction and characterization of regulated L-arabinose-inducible broad host range expression vectors in Xanthomonas. FEMS Microbiol Lett 1999, 181:217-223.
4.	Wurst H, Shiba T, Kornberg A: The gene for a major exopolyphosphatase of Saccharomyces cerevisiae. J Bacteriol 1995, 177:898-906.
5.	Lichko L, Pestov N, Kulakovskaya T, Kulaev I: Effect of PPX1 inactivation on the exopolyphosphatase spectra in cytosol and mitochondria of the yeast Saccharomyces cerevisiae. Biochemistry (Mosc) 2003, 68:740-746.
6.	Chávez F, Lünsdorf H, Jerez C: Growth of polychlorinated-biphenyl-degrading bacteria in the presence of biphenyl and chlorobiphenyls generates oxidative stress and massive accumulation of inorganic polyphosphate. Appl Environ Microbiol 2004, 70:3064-3072.
7.	Rashid M, Kornberg A: Inorganic polyphosphate is needed for swimming, swarming, and twitching motilities of Pseudomonas aeruginosa. Proc Natl Acad Sci U S A 2000, 97:4885-4890.
8.	O'Toole G, Pratt L, Watnick P, Newman D, Weaver V, Kolter R: Genetic approaches to study of biofilms. Methods Enzymol 1999, 310:91-109.
